# War Equipment

**Published:** 1915-10-16

**Authors:** 


### WAR EQUIPMENT.

### FIRST-AID PACKETS.

A correspondent who has examined the official first-aid packet issued to the English Navy side by side with that supplied to the French Army sends us a note comparing the two. The French packet appears to be of the more compact and useful shape, being 4½ in. by 3½ in. and ¾ in thick. The English packet is 3¼ in. by 2⅞ in. and 1⅛ in. thick—a more lumpy packet. The exterior of each is a cotton bag, the inevitable khaki in our own Service and the grey colour in the French. The instructions seem most explicit and concise, thus—

Naval Medical Service.

Open bag by pulling out the long threads.
Open inside packet by pulling tongues apart.
Apply in following order to the wound:
1st. Wool pad.
2nd. Gauze pad and bandage.
3rd. Waterproof.
4th. Fasten lightly with bandage.
If two wounds, put the wool pad on one, and the gauze pad on the other.

Finally there is the makers' name and the date. The French directions for use are almost identical with those for the British Navy, with the exception that one item "L'Imperméable" has been obliterated with a rubber stamp bearing the words "A ne pas utiliser." The contents of the English packet, which opens very easily, are a pad of antiseptic gamgee (probably cyanide), 2½ in. square, a six-yard bandage, white open-wove, 2½ in. wide, with a square of cyanide gauze fastened in the middle. Each end of the bandage is rolled inwards. The waterproof is used as the internal package, enveloping the dressings, and is a single-proofed batiste. The French packet differs in only a small degree. The gauze pads and gamgee are slightly larger, and they are both white and evidently medicated with boric acid. The bandage is rolled flat and separate from the gauze pad, and, as a final touch of completeness, two safety pins are added: two sheets of waterproof cover the whole. In addition to this packet we believe each soldier in the French Army is provided with an ampoule of iodine solution.

